# A JAZ Protein in *Astragalus sinicus* Interacts with a Leghemoglobin through the TIFY Domain and Is Involved in Nodule Development and Nitrogen Fixation

**DOI:** 10.1371/journal.pone.0139964

**Published:** 2015-10-13

**Authors:** Yixing Li, Meng Xu, Ning Wang, Youguo Li

**Affiliations:** 1 State Key Laboratory of Agricultural Microbiology, Huazhong Agricultural University, Wuhan 430070, People’s Republic of China; 2 Guangxi Experiment Centre of Science and Technology, Guangxi University, Nanning 530004, People’s Republic of China; 3 College of Animal Science and Technology, Guangxi University, Nanning 530004, People’s Republic of China; Institute of Genetics and Developmental Biology, CAS, CHINA

## Abstract

Leghemoglobins (Lbs) play an important role in legumes-rhizobia symbiosis. Lbs bind O_2_ and protect nitrogenase activity from damage by O_2_ in nodules, therefore, they are regarded as a marker of active nitrogen fixation in nodules. Additionally, Lbs are involved in the nitric oxide (NO) signaling pathway, acting as a NO scavenger during nodule development and nitrogen fixation. However, regulators responsible for Lb expression and modulation of Lb activity have not been characterized. In our previous work, a Jasmonate-Zim-domain (JAZ) protein interacting with a Lb (AsB2510) in *Astragalus sinicus* was identified and designated AsJAZ1. In this study, the interaction between AsJAZ1 and AsB2510 was verified using a yeast two-hybrid system and *in vitro* Glutathione S-transferase (GST) pull-down assays, resulting in identification of the interaction domain as a TIFY (previously known as zinc-finger protein expressed in inflorescence meristem, ZIM) domain. TIFY domain is named after the most conserved amino acids within the domain. Bimolecular fluorescence complementation (BiFC) was used to confirm the interaction between AsJAZ1 and AsB2510 in tobacco cells, demonstrating that AsJAZ1-AsB2510 interaction was localized to the cell membrane and cytoplasm. Furthermore, the expression patterns and the symbiotic phenotypes of AsJAZ1 were investigated. Knockdown of *AsJAZ1* expression via RNA interference led to decreased number of nodules, abnormal development of bacteroids, accumulation of poly-x-hydroxybutyrate (PHB) and loss of nitrogenase activity. Taken together, our results suggest that AsJAZ1 interacts with AsB2510 and participates in nodule development and nitrogen fixation. Our results provide novel insights into the functions of Lbs or JAZ proteins during legume-rhizobia symbiosis.

## Introduction

Hemoglobins (Hbs) have been found in humans, animals, plants and microbes. Plant Hbs include nonsymbiotic, symbiotic, and truncated Hbs [1]. Symbiotic Hbs, including leghemoglobins (Lbs) in legumes and some Hbs in actinorhizal plants, are highly expressed in root nodules and play an important role in symbiotic nitrogen fixation [2]. Non-symbiotic Hbs (nsHbs) which are classified as nsHb-1 or nsHb-2 according to their phylogeny affinity to oxygen (O_2_) [3], are widely distributed in plants, including legumes. nsHb-1s with an extremely high affinity to O_2_ appear to catalyze nitric oxide (NO)-related detoxification and regulate the NO level during legume-rhizobia symbiosis [4, 5], whereas nsHb-2s have lower O_2_ affinity and supply O_2_ to developing tissues [6]. Truncated Hbs have low affinity to O_2_ and may regulate O_2_ delivery [7].

During symbiotic nitrogen fixation by legume-rhizobia symbiosis, nitrogenase is essential for the nitrogen-fixiation by bacteroids, and functions efficiently in the microaerobic condition [1]. Lbs are a relatively large family of genes in legumes and are critical for maintaining nitrogenase activity [8]. Three Lbs have been identified in the root nodules of *Lotus japonicus* [9],while 8 Lbs have been identified in *Glycine max* root nodules [10]. All Lbs are symbiosis-specific genes that are expressed at high levels in root nodules [11, 12], but each Lb has a distinct temporal expression pattern. Lbs have been found in the cytoplasm of nodule cells at millimolar concentrations and have 2 primary functions:ⅰ) reduction of free oxygen levels to nanomolar concentrations and ⅱ) transportation of oxygen to mitochondria and bacteroids to maintain respiration which occurs at a high level in bacteroids [13].

Early research on Lbs demonstrated their ability to transport O_2_, but their important functions in nitrogen fixation have been identified only recently by using RNA interference (RNAi) to generate plants with reduced or abolished Lb expression. RNAi-mediated Lb silencing in *L*. *japonicus* increased free oxygen in root nodules, decreased the ATP/ADP ratio, and abolished nitrogenase activity [2]. Furthermore, Lbs have been shown to contribute to ROS generation in functional nodules [14]. Transcriptional analysis of plant and rhizobium genes in control and Lb-RNAi *L*. *japonicus* nodules indicated that Lbs influenced bacteroid and plant cell differentiation [15].

The recent identification of the nitrosyl-leghemoglobin (Lb-NO) complex demonstrated the capacity of Lb as a NO scavenger [16]. In root nodules, only 20% of Lb isoxygenated (oxyLb), while 80% is deoxygenated (deoxyLb) [17]. It has been proposed that deoxyLb, like nsHbs, can modulate NO levels during rhizobia-legume symbiosis by binding NO with high affinity [18]. Additionally, oxyLb can also scavenge peroxynitrite[19].

Although the functions of Lbs have been confirmed, the molecular mechanisms regulating their expression and activity remain unknown. In a previous work, we identified a TIFY (also called zinc-finger protein expressed in inflorescence meristem, ZIM) protein (designated AsJAZ1) [20] that interacted with a Lb (AsB2510, DQ199647.1) in *Astragalus sinicus* [21] by screening potential candidates with a yeast 2-hybrid system. In this study, the interaction between AsJAZ1 and AsB2510 was studied *in vivo* and *in vitro*, while symbiotic phenotypes of AsJAZ1 silencing were investigated under the condition of *A*. *sinicus*–*Mesorhizobium huakuii* 7653R [22] symbiosis. Our findings suggest that AsJAZ1 interacts with AsB2510 and participates in nodule development and nitrogen fixation.

## Materials and Methods

### Plant material and growth conditions

*A*. *sinicus* seeds collected from Xinyang, China [23]were germinated on medium with 0.5% sucrose and 1.2% agar [24] after surface-sterilization by 70% ethanol for 5min and 2% sodium hypochlorite for 10 min. Germinated seeds were transferred to pots filled with sterile sand and watered with nitrogen-free Fahraeus solution (NFS)[25]. For plant inoculation, *M*. *huakuii* 7653R was cultured in liquid TY medium at 28°C with shaking for 24–36 h until an optical density at 600 nm (OD_600_) reached 1.0. The bacteria were collected by centrifugation, washed three times with 0.9% NaCl and re-suspended in NFS. After cotyledon expansion, the plantlets were flood-inoculated with 1mL (approximately 1 ×10^7^ CFU/mL) of *M*. *huakuii* 7653R culture. All plants were grown in a greenhouse at 24°C (day)/18°C (night) with a 16-h/8-h day/night cycle. Roots, stems, leaves and nodules were harvested on different days post inoculation and frozen in liquid nitrogen.

### Bacterial and yeast strains

*Escherichia coli* strain Rosetta (DE3) [26], which contains pRARE (argU, argW, ileX, glyT, leuW, proL) (Cm^r^), was used as the expression system for recombinant proteins. *Saccharomyces cerevisiae* strainAH109 [27] (*MATa*, *trp1-901*, *leu2-3*, *112*, *ura3-52*, *his3-200*, *gal4Δ*, *gal80Δ*, *LYS2*:: *GAL1*_*UAS*_ *–Gal1*_*TATA*_ *–His3*, *GAL2*_*UAS*_ *–Gal2*_*TATA*_ *–Ade2*, *URA3*:: *MEL1*_*UAS*_ *–Mel1*_*TATA*_ *-lacZ*, *MEL1*) and *S*. *cerevisiae* strain Y187 [28] (*MATα*, *ura3-52*, *his3-200*, *ade2-101*, *trp1-901*, *leu2-3*, *112*, *gal4Δ*, *gal80Δ*, *URA3*:: *GAL1*_*UAS*_ *–Gal1*_*TATA*_ *–lacZ*, *MEL1*) were used for the yeast 2-hybrid assays.

### Amplification of full-length cDNA and plasmid construction

**Fig 1:**
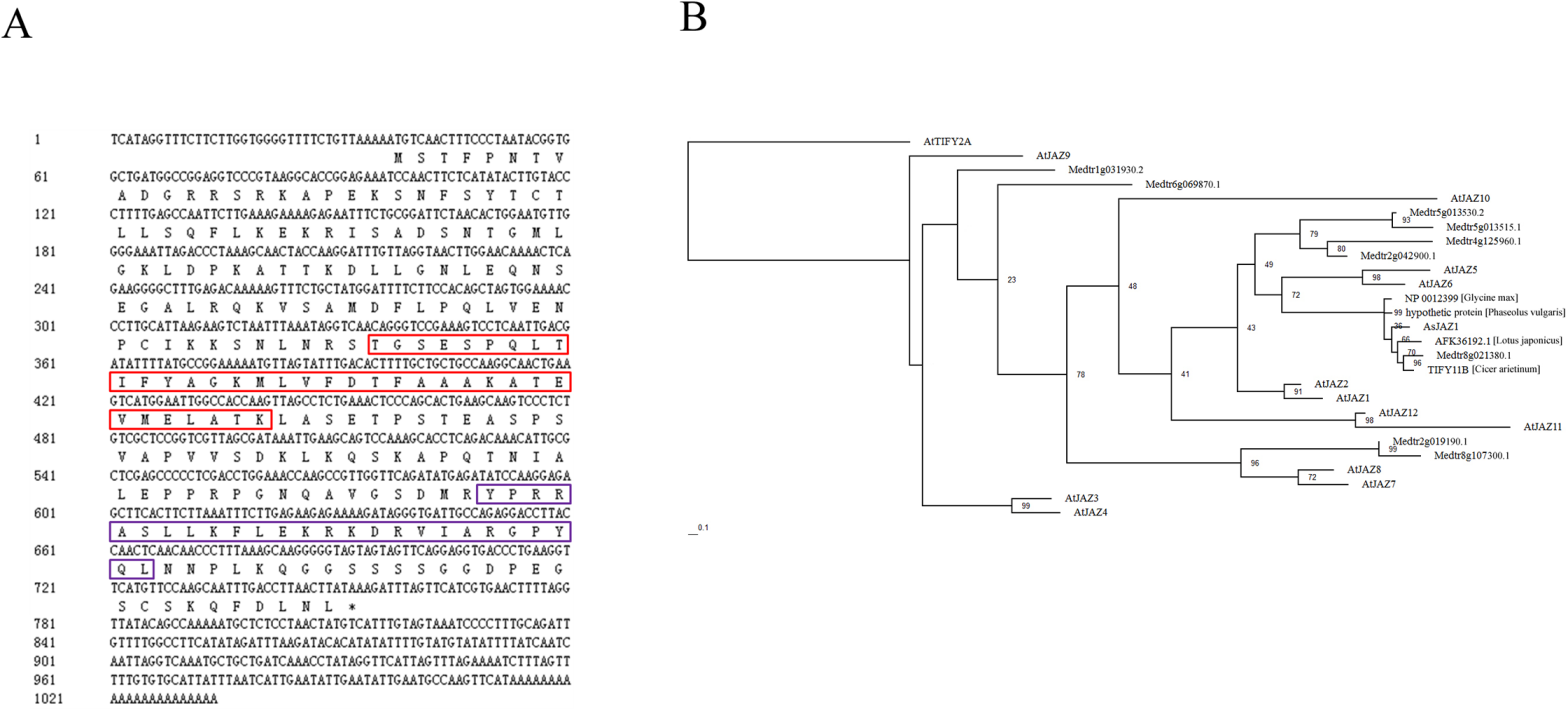
AsJAZ1 is a putative JAZ protein. **(**A) Deduced amino acid sequence of AsJAZ1. Conserved motifs are boxed. The red box indicates the TIFY domain and the purple box indicates the Jas domain. (B) Maximum likelihood phylogenic tree constructed by RAxML software version 8.0.20 displaying the relationship among AsJAZ1 and JAZs in *A*. *thaliana* (data collected from TAIR, http://www.arabidopsis.org), *M*. *truncatula* (data collected from *M*. *trucatula* database http://www.jcvi.org/medicago) and 4 homologous proteins in other leguminous plants (data collected by NCBI BLASTP search). Bootstrap values (%) were calculated by 500 resampling repetitions and AtTIFY2A, which is a member of ZML subfamily, was selected as the outgroup sequence.

The Jasmonate-Zim-domain (JAZ) gene fragment originally obtained using the yeast 2-hybrid system did not contain a full coding region. In order to obtain the full-length gene sequence, 5′ and 3′ random amplification of cDNA ends (RACE) was performed using the BD SMART RACE cDNA Amplification Kit (Clontech). Nodules were collected on the 35th day after inoculation and total RNA was extracted by Trizol reagent (Invitrogen). 5′ and 3′ RACE was carried out according to the manufacturer’s instructions. The complete coding sequence obtained by RACE was designated *AsJAZ1* (Fig 1A). For RNAi, a 362-bp fragment including the 3′- UTR region and a 102-bp 3′-terminal coding region of *AsJAZ1* was amplified by PCR. To generate the pRNAi-JAZ1 vector, the sense PCR product was inserted between the *Sma*I and *Bam*HI sites in the pCAMBIA1301-35S-int-T7 plasmid [29], whereas the antisense product was inserted between the *Xba*I and *Pst*I sites. The PCR primers used in this work are listed in supporting information.

### Sequence analysis and phylogenetic tree construction

Conserved domains in AsJAZ1 were analyzed using the Pfam database (http://pfam.sanger.ac.uk) [30]. To investigate the phylogenetic relationships among the members of the JAZ subfamily in *Arabidopsis thaliana* and several leguminous plants, a maximum likelihood (ML) phylogenic tree was constructed. The full-length amino acid sequences of 12 JAZs from *A*. *thaliana* and 9 JAZs from *Medicago truncatula* were downloaded from the TAIR database (http://www.arabidopsis.org) and *M*. *truncatula* database (http://www.jcvi.org/medicago), respectively. Some other homologous proteins of AsJAZ1 in leguminous plants were obtained by NCBI BLASTP search. Sequence alignments were performed by ClustalX 2.0 [31], after which ProtTest 2.4 was used to select the best matrix (JTT model) for the ML tree. The ML tree was generated using the RAxML version 8.0.20 [32] software. Bootstrap values (%) were calculated by 500 resampling repetitions.

### Protein–protein interaction in yeast cells

*AsB2510* cDNA was amplified by PCR (primers are listed in S1 Table) and inserted between the *Bam*HI and *EcoR*I sites of pGBKT7 (TRP1, Kan^r^) (Clontech, USA). Four deletion mutants of *AsJAZ1*were constructed to determine the domain(s) interacting with AsB2510. AsJAZ1-1 (13–213), AsJAZ1-2 (13–186), AsJAZ1-3 (13–135) and AsJAZ1-4 (13–102) fragments were amplified by PCR and inserted between the *Bam*HI and *Eco*RI sites of the pGADT7 plasmid (LEU2, Amp^r^) (Clontech). The *S*. *cerevisiae* AH109 strain containing the complete AsJAZ1 gene and the 4 deletion mutant strains were hybridized with *S*. *cerevisiae* Y187 harboring AsB2510. The interaction between mammalian p53 and SV40 served as a positive control, whereas the interaction of lamin (Lam) with SV40 served as a negative control. The operations were performed according to the methods described in the Clontech yeast protocols handbook and by Chen et al. [29]. Briefly, yeast cells were selected on synthetic dextrose plates (SD/-Leu-Trp-His-Ade) with 80 mg/mL 5-Bromo-4-chloro-3-indolyl β-D-galactopyranoside (X-gal). Positive cells grown in liquid selection medium were collected and washed twice with Z-buffer (yeast protocols handbook, Clontech), resuspended in 100 mL Z-buffer, and permeabilized via 3 freeze-thaw cycles in liquid nitrogen and a 37°C water bath. The resulting cell extracts were added to 0.7 mL Z-buffer supplemented with 50 mmol/L β-mercaptoethanol and 160 μL 4 mg/mL o-nitrophenyl-β-D-galactopyranoside (ONPG) and incubated at 30°C until the reaction mixture became yellow, at which point the reaction was terminated by the addition of 0.4 mL of 1.0 mol/L Na_2_CO_3_. The reaction mixture was centrifuged for 10 min at 14,000× *g*, after which β-galactosidase activity was measured in the supernatant by a spectrophotometer at a wavelength of 420 nm.

### *In vitro* Glutathione S-transferase (GST) pull-down assay

*AsJAZ1* amplified by PCR was inserted between the *Nde*I and *Bam*HI sites of pET28a(+) (Novagen, Germany) to allow expression of an His-AsJAZ1 fusion protein. After induction by 1 mmol/L isopropyl β-D-1-thiogalactopyranoside (IPTG) at 37°C for 4 h, the bacteria cells were disrupted using a sonicator. His-AsJAZ1 was purified from the lysate using a nickel-agarose column (Bio-Rad, USA) according to the manufacturer’s instructions. For GST-AsB2510 fusion proteins expression, the coding sequence of AsB2510 was digested by *Bam*HI and *Xho*I and inserted into a pGEX-6p-1 vector (GE Healthcare, Germany), generating the recombinant plasmid pGEX-6p-1-Lb. *E*. *coli* Rosetta (DE3) (Novagen) harboring pGEX-6p-1-Lb was induced with 1mmol/L IPTG for 12 h at 20°C. The GST pull-down assay was conducted as described by Zhu et al. [33]. The GST-AsB2510 fusion protein was purified and bound to the glutathione Sepharose 4B column. GST beads alone or binding with GST-tag were used as negative control samples. The purified His-AsJAZ1 fusion protein was incubated with the immobilized GST-AsB2510 fusion protein or the control samples in 1 mL of interaction buffer (20 mmol/L Tris-HCl, 100 mmol/L KCl, 2 mmol/L MgCl, 5% glycerol, pH 8.0) for 1 h at 4°C with shaking. After the reaction, the beads were washed 3 times with PBS (2.7 mmol/L KCl,140 mmol/L NaCl,1.8 mmol/L KH_2_PO_4,_10 mmol/L, Na_2_HPO_4_, pH 7.4) containing 1% Triton X-100, followed by 3 washes in PBS. The protein complexes were separated via SDS-PAGE and His-AsJAZ1 was detected using anti-His-tag antibody.

### Bimolecular fluorescence complementation assay

The bimolecular fluorescence complementation (BiFC) assay was carried out using the method described by Kang et al. [34]. The full-length cDNA of the AsB2510 gene was amplified by PCR (primers in S1 Table) and inserted between *Bam*HI and *Kpn*I sites of pSCYNE [35] to obtain AsLb: SCFPN (N-terminal Split Cyan Fluorescent protein). The coding region of AsJAZ1 was cloned into pSCYCE-R [35] to obtain AsJAZ1:SCFPC (C-terminal Split Cyan Fluorescent protein). The *AsJAZ1*△*tify* coding region was cloned into pSCYNE to obtain AsJAZ1△TIFY: SCFPC. *Agrobacterium tumefaciens* GV3101 (pMP90) strains were transformed respectively with the constructs above, cultured in Luria-Bertan (LB) liquid medium, collected by centrifugation, and re-suspended in infiltration buffer. *A*. *tumefaciens* GV3101 cells containing different paired plasmids were mixed and adjusted OD_600_ to 0.5. After incubation for 2 to 4 h at room temperature, the *A*. *tumefaciens* GV3101 mixture was injected into the leaves of 6-week-old *Nicotianaben thamiana* plants. Two to 3 days after the injection, CFP fluorescence was observed using a Zeiss LSM510 laser scanning microscope. Wild-type tobacco plants were grown in a growth chamber at 22°C and 70% relative humidity under a 16-h-light/8-h-dark photoperiod for approximately 6 weeks.

### Real-time RT-PCR

Total RNA was extracted from each sample using Trizol reagent (Invitrogen, USA) according to the manufacturer’s protocol. DNaseⅠ (Takara, Japan) was used to remove genomic DNA, followed by RNA purification with phenol:chloroform. RNA purity was assessed by calculating the OD_260_/OD_280_ ratio of each sample. The RNA concentration was measured at 260 nm. An aliquot of 1μg total RNA was used for reverse transcription. First-strand cDNA was synthesized by RevertAid Reverse Transcriptase (Fermentas, USA) using oligo(dT)_18_ primers. Real-time PCR was performed with the SYBR Premix ExTaqII (Takara, Japan). The data were analyzed by the 2^-ΔΔCt^ method with *Asactin* as the reference gene [36]. Each experiment was performed in triplicate.

### Plant transformation

*Agrobacterium rhizogenes* K599 strains containing pRNAi-JAZ1 or empty pCAMBIA1301-35S-int-T7 were cultured in 50 mL of LB medium until OD_600_ reached approximately 1.0. Plant transformation was performed by the method of Wang et al. [37]. Sterilized 7-day-old *A*. *sinicus* seedlings were cut in the middle of the hypocotyl and immersed in the K599 culture for 10 min. The seedlings were blotted with sterilized filter paper, placed on Murashige and Skoog (MS) [38] solid basal medium and grown in a growth chamber maintained at 22°C with a 16-h-light/8-h-dark light cycle for co-cultivation. After 3 days, the explants were transferred to fresh MS medium with 500 mg/L carbenicillin (Cb). For selection of transgenic hairy roots, root tips (2 to 3 mm) were excised and stained by X-gluc overnight at 37°C. The remaining portion of the hairy roots was labeled. Two or 3 transgenic hairy roots with GUS-positive root tips were kept for each seedling. Seedlings harboring transgenic hairy roots were planted in pots with sterile sand and watered with NFS. All plantlets were grown in a growth chamber maintained at 22°C with a 16h-light/8h-dark light cycle. The plants were inoculated with *M*. *huakuii* 7653R after 3 days of nitrogen starvation.

### Nitrogenaseactivity assay

Nitrogenase activity was assessed by acetylene reduction activity method [39]. For each sample, 9 hairy root lines were analyzed. Every three hypogeal parts of hairy root plants (including nodules and roots) were incubated in 2 mL of acetylene for 2 h at 28°C in 20-ml glass bottles with rubber seals. The amount of ethylene was measured using an East & West Analytical Instrument GC 4000A gas chromatograph.

### Microscopic analysis

Light and transmission electron microscopy (TEM) were carried out with the protocols described as Li et al. [23]. Total 12 control and 11 RNAi nodules collected from 3 repetitions have been analyzed. Paraffin-embedded nodule sections were stained with toluidine blue before observation by light microscopy. Ultrastructure observation was performed by TEM (HITACHI, H-7650).

### Statistical analysis

The significance of the data was analyzed using independent-samples T test and multiple comparison was performed using one-way ANOVA method by SPSS 13.0. Bars in figures represent the SE (standard error) of three independent experiments. The data in Table 1 represent mean±SE of three independent experiments.

**Table 1:** Biomass of plantlet and nodule and nodule nitrogenase activities.

Sample	Fresh weight per plantlet (g)	Nodule fresh weight (mg)	Nitrogenase activity (μmol/g nodule h)
Control	0.69±0.07	0.89±0.06	5.75±0.35
RNAi	0.25±0.06*	0.68±0.02*	1.25±0.04*

Fresh weights of above-ground sections of hairy roots at 35 d after inoculation. Nodules were harvested at 35 d after inoculation. The data represent the mean ±SE of three independent experiments. Significance of the data was analyzed using independent-samples T test.

*, indicate the means in the same vertical column were significant at *P*<0.05.

## Results

### Characterization of JAZ1 isolated from *A*. *sinicus*

Full-length cDNA sequence of the protein that we obtained previously was 1,034 bp including an open reading frame of 717 bp, which encoded a protein of 239 amino acid residues. Motif analysis by Pfam software demonstrated that the encoded protein contains 2 typical conserved domains of the plant TIFY transcription factor family, the central TIFY domain and C-terminal CCT_2 (Jas) domain (Fig 1A), while no DNA binding domain was identified. Because TIFY and Jas are the 2 typical domains of JAZ subfamily, the obtained protein was designated AsJAZ1. NCBI blastp analysis showed that AsJAZ1 displayed high identity (67%-73%) in its amino acid sequence with hypothetical proteins expressed in *Phaseolus vulgaris* (XP007135415), *L*. *japonicus* (AFK36192.1), *G*. *max* (NP001239983), *M*. *truncatula* (Medtr8g021380.1), and *Cicer arietinum* (TITY 11B). ML phylogenetic analysis of AsJAZ1 protein, some putative JAZs in several leguminous plants and JAZs in *A*. *thaliana* was performed using RAxML software. As shown in the ML phylogenetic tree in Fig 1B, AsJAZ1 was closely related to its homologous proteins in legumes. Our results were similar with those produced by the ML phylogenetic analysis of AtJAZ proteins by Bai et al. [40], which indicated that AtJAZ1, AtJAZ2, AtJAZ5 and AtJAZ6 were clustered in one group. The phylogenetic analysis of AsJAZ1 also showed that it was clustered in the same group with AtJAZ1, AtJAZ2, AtJAZ5 and AtJAZ6 (Fig 1B). Therefore, based on amino acid sequence analysis, we suggest that AsJAZ1 is a member of the JAZ subfamily.

### Interaction between AsJAZ1and AsB2510 requires the TIFY domain

**Fig 2:**
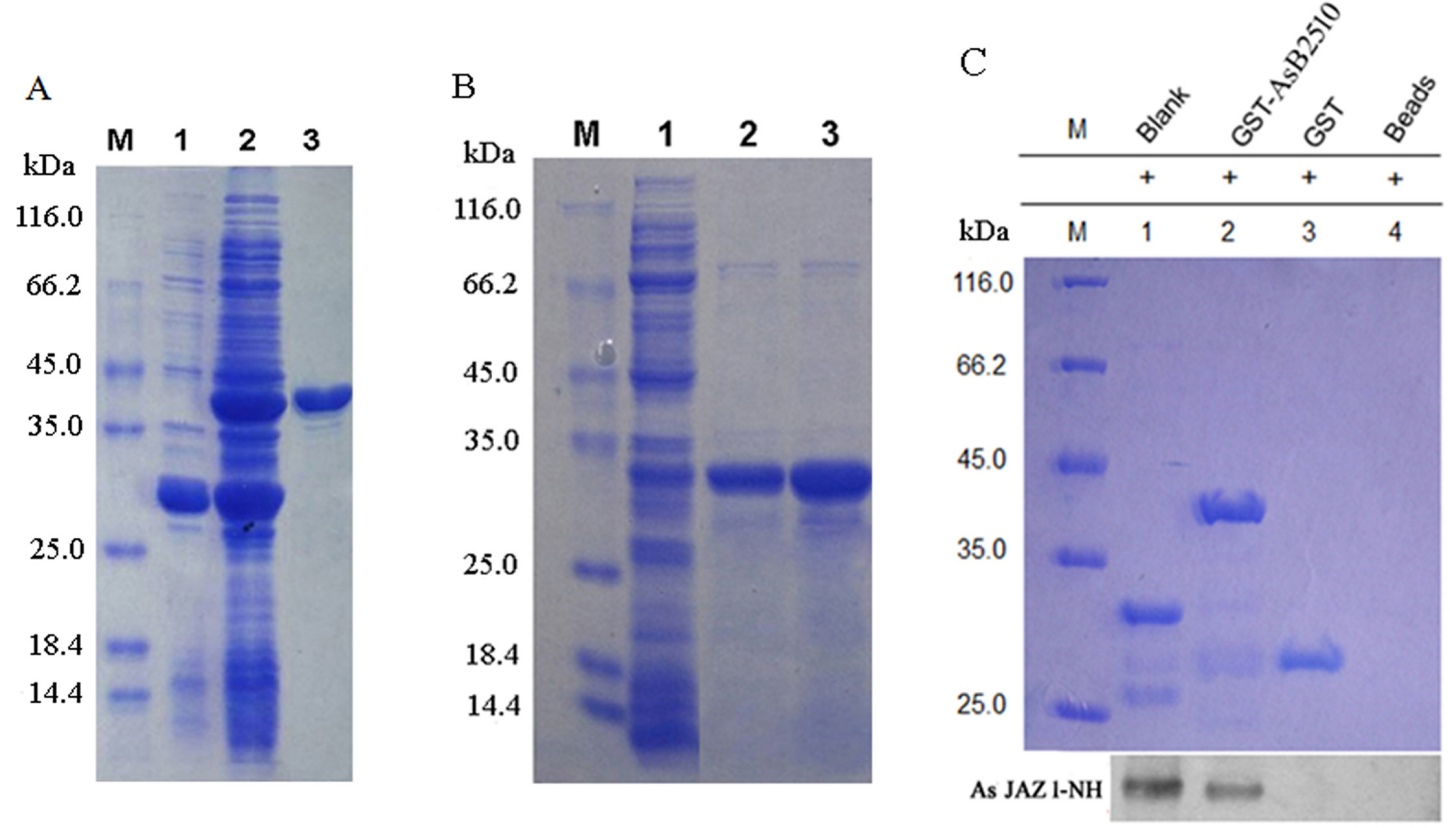
In vitro GST pull-down assay of AsJAZ1 and AsB2510. (A) Purification of GST-AsB2510 expressed in *E*. *coli* Rosetta (DE3). M: protein marker; lane 1: total protein from *E*. *coli* Rosetta (DE3) containing empty pGEX-6p-1; lane 2: total protein from *E*. *coli* Rosetta (DE3) containing GST-AsB2510; lane 3: GST-AsB2510 purified using glutathione Sepharose 4B column. (B) Purification of His-AsJAZ1 expressed in *E*. *coli* Rosetta (DE3). M: protein marker; lane 1: total protein from *E*. *coli* Rosetta (DE3) expressing His-AsJAZ1; lane 2 and lane 3: His-AsJAZ1 purified by nickel-agarose column. (C) SDS-PAGE and western blotting of the samples obtained by GST pull-down. His-AsJAZ1 incubated with either the immobilized GST tag or the beads alone served as the negative control samples. Anti-His antibody was applied for the western blot. AsJAZ1-NH, AsJAZ1 with an N-terminal His tag. Blank, purified His-AsJAZ1.

In order to verify the interaction between AsJAZ1 and AsB2510, agarose beads containing the GST-AsB2510 fusion protein (Fig 2A) were used to pull down the His-AsJAZ1 fusion protein (Fig 2B), after which AsJAZ1 bound to the beads was detected by western blotting using anti-His antibody. As shown in Fig 2C, when His-AsJAZ1 was incubated with the immobilized GST-AsB2510, it was detected in the eluent by anti-His-tag antibody, while no hybridization signal was observed in the negative control samples (His-AsJAZ1 incubated with GST tag or the Beads alone). These results demonstrated that AsB2510 could pull down AsJAZ1, indicating that the two proteins interacted directly *in vitro*.

**Fig 3:**
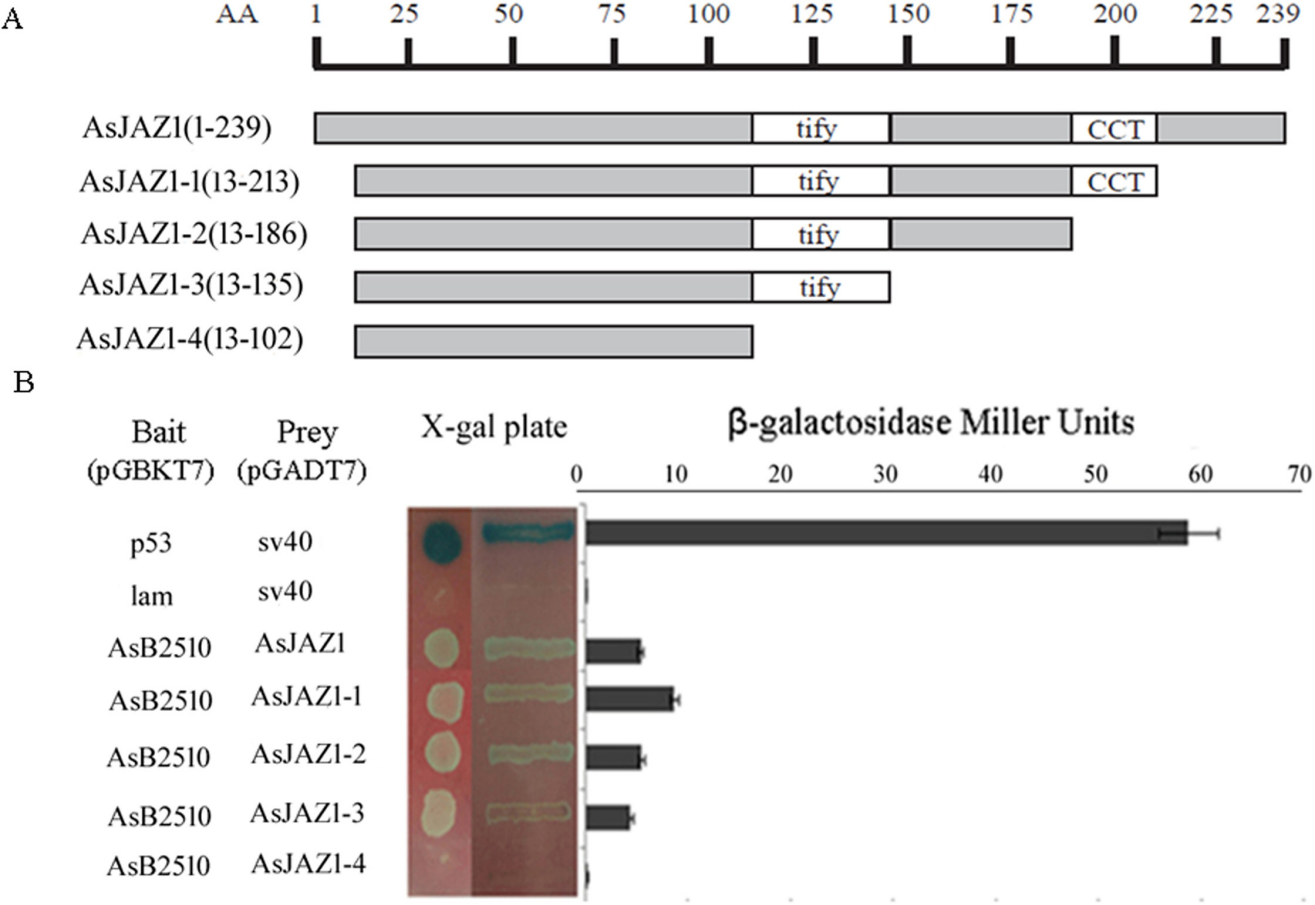
Interaction between AsJAZ1 and 4 AsJAZ1 derivatives with AsB2510 in yeast cells. **(**A) Construction of the 4 AsJAZ1 derivatives. (B) The strength of the interaction was detected using a β-galactosidase activity assay. Yeast cells were grown on SD/-Leu-Trp-His-Ade plates containing 80 mg/L X-Gal.

To further characterize the critical domain involved in binding of AsJAZ1 to AsB2510, the coding regions of AsJAZ1 and its four derivatives (Fig 3A) were separately inserted into pGADT7 and introduced into *S*. *cerevisiae* AH109 to allow assessment of their interactions with AsB2510 via yeast 2-hybrid system. The *S*. *cerevisiae* AH109 groups harboring the constructs were hybridized with *S*. *cerevisiae* Y187 (containing AsB2510) and galactosidase activity was examined. As shown in Fig 3B, positive galactosidase activity was observed in the supernatant samples from the yeast strains with AsJAZ1 (1–239), AsJAZ1-1 (13–213), AsJAZ1-2 (13–186) and AsJAZ1-3 (13–135) constructs, indicating that interaction between AsB2510 and AsJAZ1 occurred when the TIFY domain was present. However, the presence or absence of Jas domain barely influenced the interaction between AsB2510 and AsJAZ1. The supernatant from the yeast stain with the AsJAZ-4 (13–102) construct, without the TIFY domain, displayed no galactosidase activity and no growth of relative yeast cells was observed on the exposed SD/-Trp-Leu-His-Ade+X-gal plate. These results indicate that the TIFY domain is indispensable for the interaction of AsJAZ1 with AsB2510. Moreover,as shown in Fig 3B, the 13 amino acid residues at the N-terminal of AsJAZ1 negatively affected its interaction with AsB2510.

### Interaction between AsTIFY1 and AsB2510 occurs in living tobacco cells

**Fig 4:**
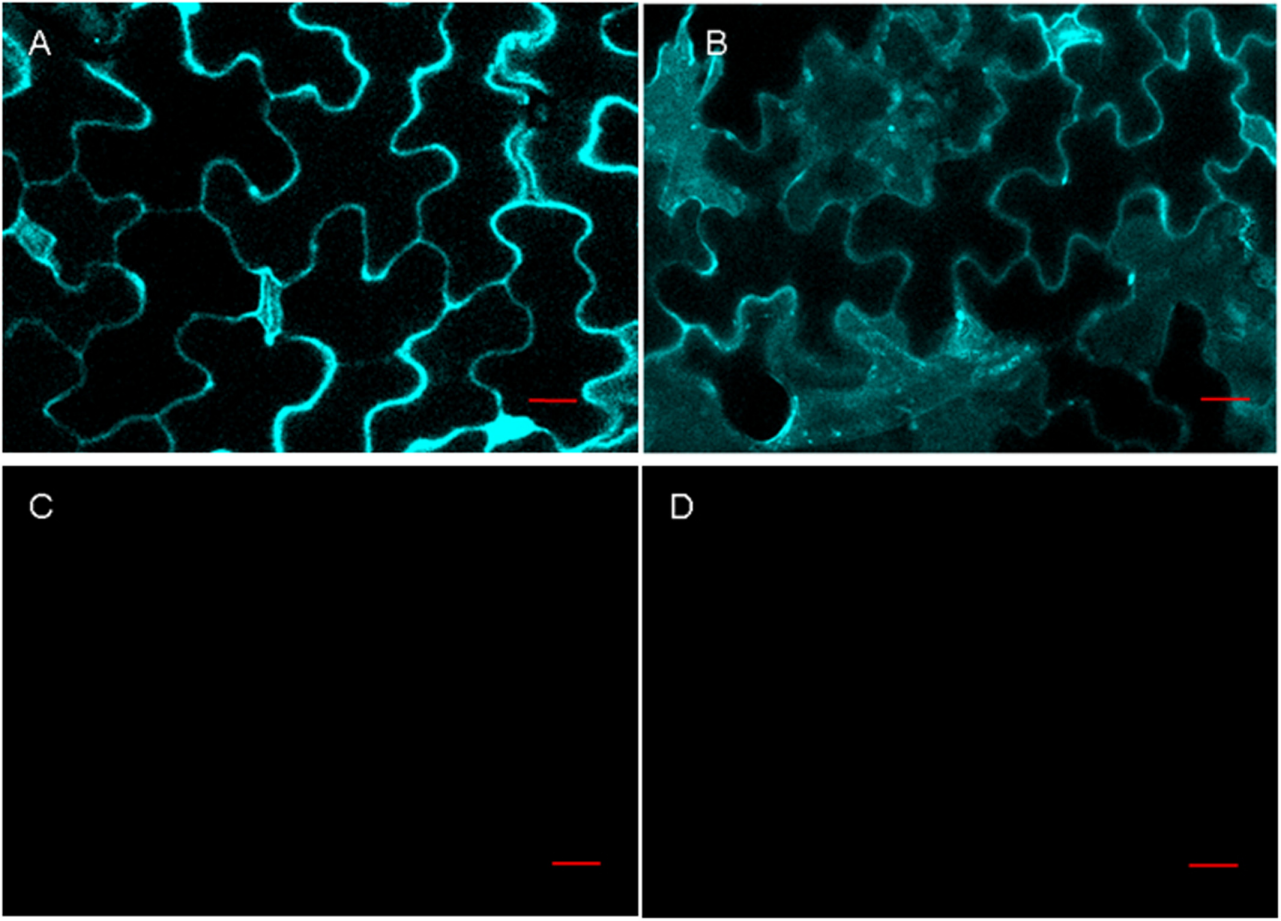
The interactions of AsJAZ1 and AsJAZ1ΔTIFY with AsB2510 were identified by BiFC. CFP fluorescence was observed in tobacco leaf epidermal cells using a confocal laser microscope. (A) CBL1 fluorescence with CIPK24 (positive control). (B) AsJAZ1 fluorescence with AsB2510. (C) No fluorescence was observed for AsJAZ1ΔTIFY with AsB2510. (D) No fluorescence was observed for CBL10 and CIPK24 (negative control). Bars = 20 μm.

The interaction of AsB2510 and AsJAZ1 in plant cells was confirmed using BiFC. AsJAZ1 and AsB2510 were respectively fused with the C terminal and N terminal of SCFP, and their interaction was observed in the epidermal cells of tobacco leaf. Fluorescence was observed on the cell membrane and in cytoplasm when AsB2510 and AsJAZ1 were co-transformed (Fig 4B), indicating that AsB2510 and AsJAZ1 interacted on the cell membrane and in cytoplasm in tobacco cells. Consistent with the results obtained in yeast cells, AsB2510 and AsJAZ1△TIFY did not interact in tobacco cells (Fig 4C). In addition, no interaction was observed between calcineurin B-like protein (CBL) 10 and CIPK (CBL interacting protein kinase) 24 (negative control) (Fig 4D), whereas interaction between CBL1 and CIPK24 (positive control) was observed (Fig 4A).

### Expression patterns of AsB2510 and AsJAZ1 genes under symbiotic conditions

**Fig 5:**
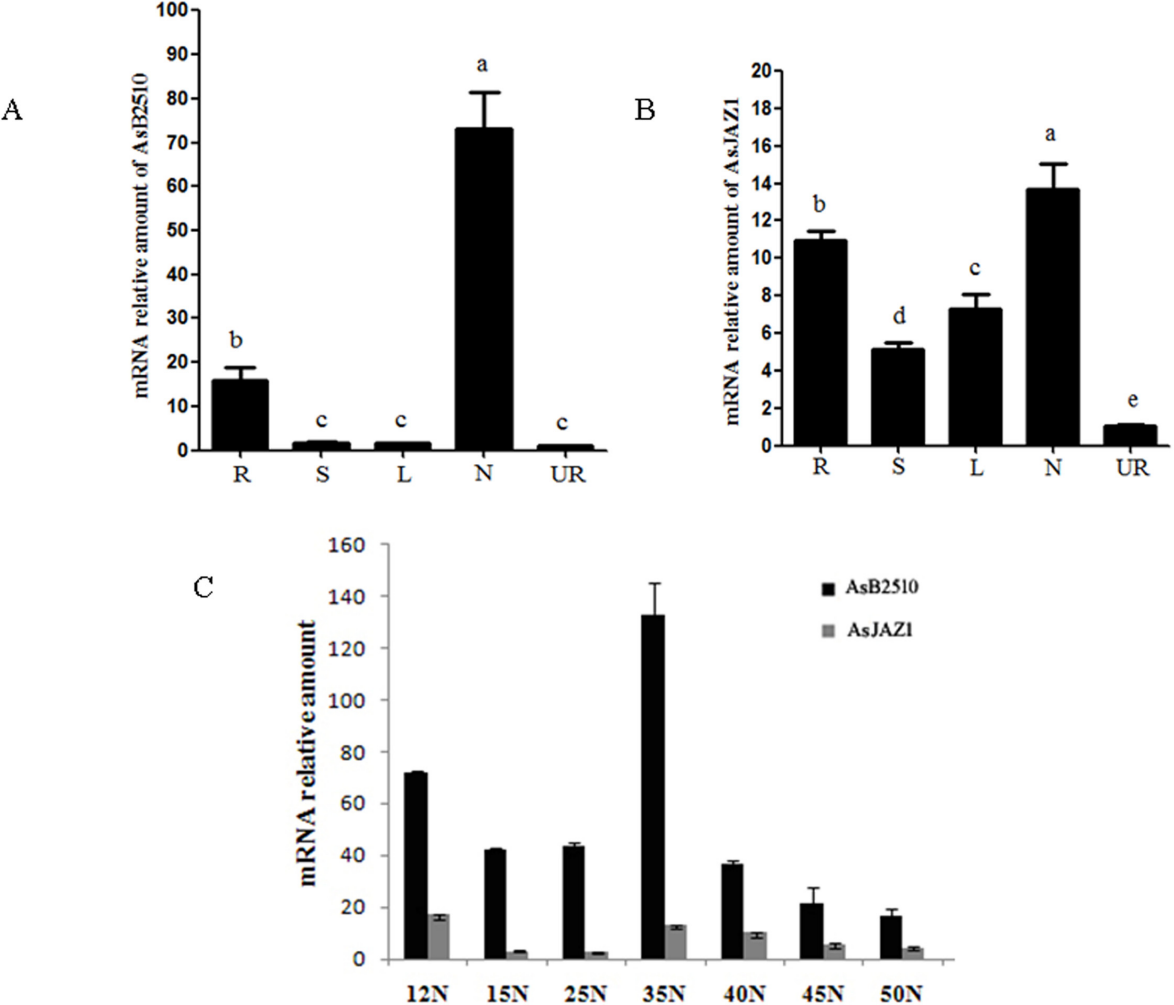
Expression patterns of *AsB2510* and *AsJAZ1*. (A) and (B) Tissue-specific expression levels of *AsB2510* and *AsJAZ1* in roots (R), stems (S), leaves (L), and nodules (N) 22 d after inoculation with *M*. *huakuii* 7653R. Uninoculated roots (UR) served as the control sample. (C) Temporal expression patterns of *AsB2510* and *AsJAZ1* in nodules harvested on different days after inoculation. 2^-ΔΔCt^ method was used to analyze the data and *Asactin* served as the reference gene. The experiment was performed in triplicate and bars represent the SE of three independent experiments. One-way ANOVA was used for multiple comparison and different characters indicate the means were significant at *P*<0.05.

Semi-quantitative analysis by Chou et al. showed that AsB2510 was highly expressed in nodules [21]. Here we used real-time RT-PCR to determine the tissue expression patterns of AsB2510 and AsJAZ1 (Fig 5A and 5B). On the 22nd day post inoculation with *M*. *huakuii* 7653R, *AsB2510* and *AsJAZ1* were both up-regulated in inoculated roots in comparison with their levels in un-inoculated roots. *AsB2510* was expressed in nodules and inoculated roots, but the highest transcript level was observed in nodules. *AsJAZ1* showed higher expression levels in nodules and roots, but was expressed in shoots and leaves as well, in contrast to *AsB2510*. In a previous work, Chou et al. reported that AsB2510 was expressed at a high level in infected roots and nodules [21], which was consistent with the results of the present study.

The temporal expression patterns of *AsB2510* and *AsJAZ1* were analyzed in nodules on different days following inoculation with *M*. *huakuii* 7653R (Fig 5C). The high transcript level of *AsB2510* in nodules was maintained during nodule development and nitrogen fixation, after which it decreased in senescent nodules. In contrast, the *AsJAZ1* expression level was higher at the early stage of nodule development and was lower in mature nodules. These results demonstrate that rhizobia inoculation up-regulated expression of both *AsB2510* and *AsJAZ1*.

### Silencing of AsJAZ1 impairs bacteroid development and nitrogen fixation

**Fig 6:**
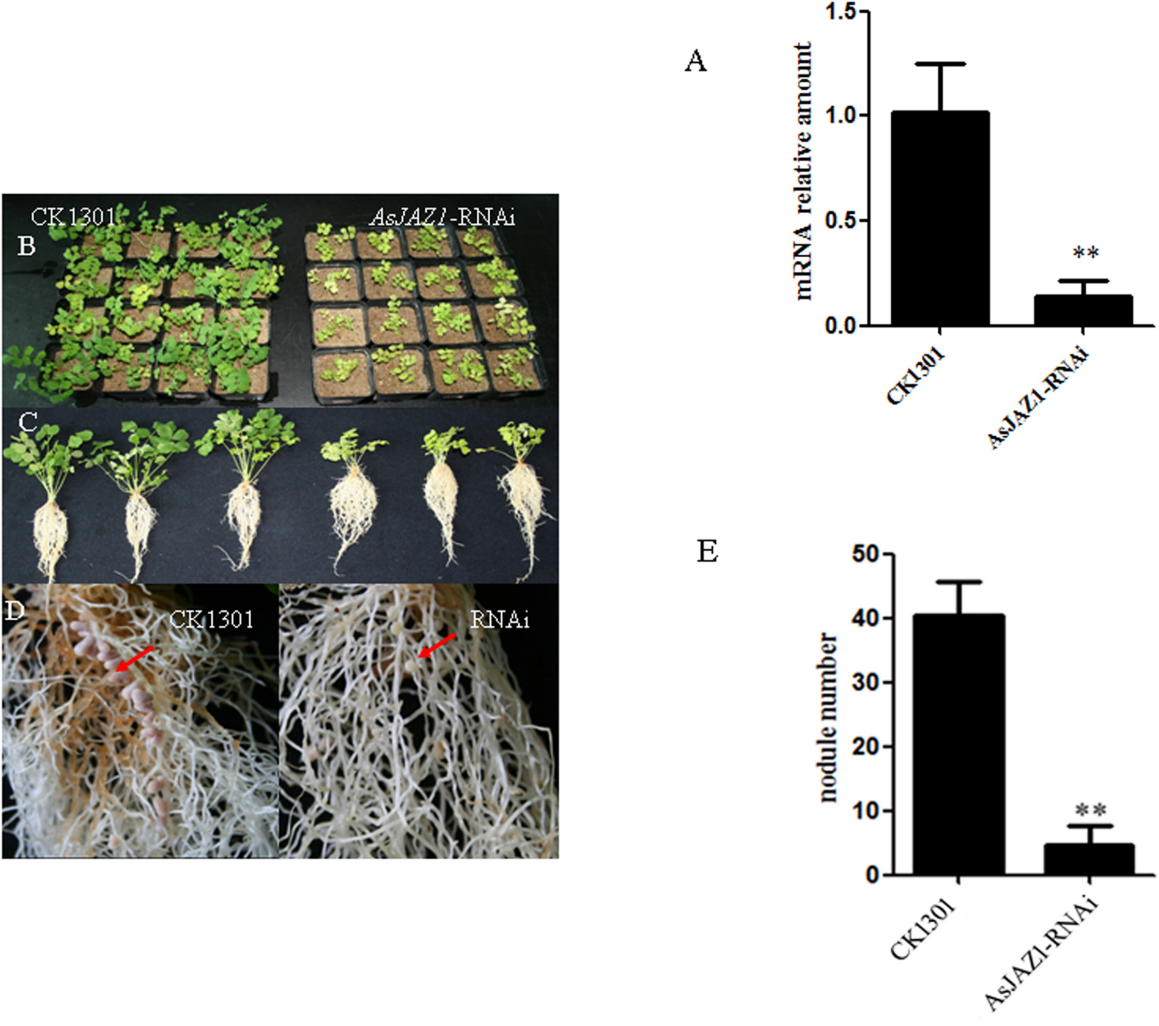
Symbiotic phenotypes associated with *AsJAZ1*-RNAi. Hairy roots and nodules with empty pCAMBIA1301-35S-int-T7 served as the control samples. The phenotype was observed 30 days after inoculation. (A) Efficiency of RNAi in hairy roots. The experiment was performed in triplicate. Nine to 10 hairy root plants were analyzed in each experiment and the total numbers of control and RNAi plantlets were 30 and 28, respectively. Bars represent the SE of three independent experiments. **, difference is significant at *P*<0.01. (B) Above-ground sections for control (left, CK1301) and RNAi hairy roots. (C) Appearances of control (left 3) and RNAi (right 3) hairy root plants. (D) Appearance of root nodules on control (left, CK1301) and RNAi hairy roots. The arrows indicate normal nodules on control roots or ineffective nodules on RNAi hairy roots. (E) Number of nodules on control and RNAi hairy roots. The experiment was performed in triplicate and bars represent the SE of three independent experiments. **, difference is significant at *P* < 0.01.

**Fig 7:**
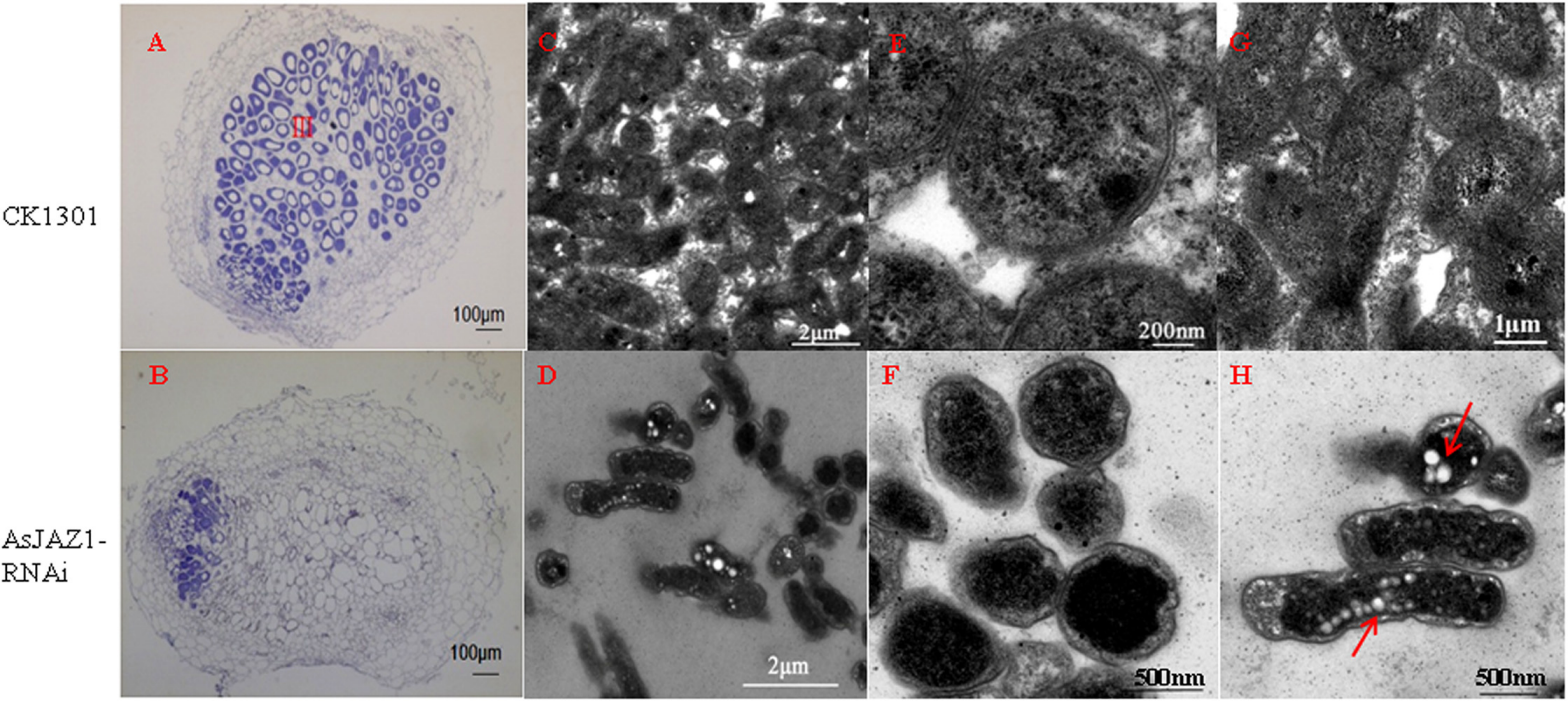
Microscopic observations of the development of nodules and bacteroids in the RNAi experiment. Nodules containing empty pCAMBIA1301-35S-int-T7 were used as the control samples. Paraffin-embedded sections of control (A) and RNAi (B) nodules (30 d after inoculation) were stained with toluidine blue and observed under a light microscope. In comparison with the control cells, much fewer rhizobia were included in the RNAi nodule cells, whereas the nitrogen-fixing zone (Ⅲ) could not be recognized in the RNAi nodules. Ultrastructural observation of the control (C) and RNAi nodules (D) was performed by TEM. Compared with the bacteroids in the control nodule cells (E), bacteria in the RNAi nodule cells showed abnormal developmental morphology (F) and contained PHB particles (arrows) (H) which were not present in functional bacteroids (G). The nodule section in (C) was from the infection zone closer to the nitrogen-fixing zone, and (E) was the magnification of some bacteroids in (C). The nodule section in (G) was from the nitrogen-fixing zone. Bars in (A) and (B), 100 μm, bars in (C) and (D), 2 μm; bars in (F) and (H), 500 nm; bars in (G), 1μm; bars in (E), 200 nm.

Transgenic hairy roots with down-regulated *AsJAZ1* expression levels were generated via *A*. *rhizogenes* K599-mediated RNAi. A 362-bp fragment including 3’-untranslated region and partly coding sequence was selected for RNAi construction. The efficiency of RNAi was assessed by real-time RT-PCR (Fig 6A). The transcript level of *AsJAZ1* was suppressed by 86.2% in RNAi hairy root plants (n = 28) in comparison with that of the control plants (n = 30) expressing the empty vector. On the 30th day post inoculation with *M*. *huakuii* 7653R, the symbiotic phenotypes were analyzed. The composite plants of RNAi roots were dwarfed and had yellow leaves, whereas the plants of control roots displayed healthy growth (Fig 6B and 6C). The fresh weights of the above-ground sections of the RNAi hairy roots (35 days post inoculation) were much lower than those of the controls roots (Table 1). On the underground sections, the RNAi roots had much fewer nodules than the control roots (Fig 6E). The RNAi nodules were small and white (Fig 6D) and had much lower nitrogenase activity than the control nodules (Table 1). In comparison with the control nodules, the RNAi nodules contained remarkably decreased numbers of rhizobia, and the nitrogen-fixing zone (Ⅲ zone) could not be recognized (Fig 7A and 7B). Furthermore, to determine whether AsJAZ1 is essential for symbiosome development, ultrastructural comparisons of the control and RNAi nodules were performed by TEM. In comparison with bacteroids in control nodules (Fig 7C and 7E), the bacteria in the RNAi nodule cells lacked the typical features of bacteroids (Fig 7D). Most bacteria exhibited abnormal morphology and failed to continued development, indicating that their differentiation was blocked in infection zone (Fig 7F). Furthermore, in some bacteria in RNAi nodule cells, many PHB particles were observed (Fig 7H) which were not present in functional bacteroids in control nodules (Fig 7G). Our results indicate a suppression of bacteroid development and nitrogen fixation in RNAi nodules. Therefore, the down-regulation of *AsJAZ1* impairs bacteroid development and nodule function, leading to ineffective nodules.

## Discussion

TIFY proteins, previously known as ZIM proteins [41] constitute a large family of transcription factors in plants that are characterized by the TIFY motif in their ZIM domain. TIFY family proteins can be classified into 4 subfamilies based on their constituent domains [40]: TIFY subfamily, containing only the TIFY (ZIM) domain; ZML (ZIM-like) subfamily, containing the TIFY, CCT and ZML domains; JAZ subfamily, containing the TIFY and Jas domains (also called the CCT_2 domain); and PPD subfamily, containing the TIFY domain and a truncated Jas domain. The TIFY family plays a role in regulating diverse biological processes in plants, including elongation of the petiole and hypocotyls [42], root growth [43], flower abscission [44], leaf development [45] and responses to abiotic and biotic stress [46–48].

Recent research has focused on the relationship between JAZs and phytohormones. JAZs regulate the expression of JA-responsive genes by acting as transcriptional repressors of the jasmonate signaling pathway [49]. Under normal physiological conditions, JAZs inhibit JA pathway by repressing the activity of transcription factor MYC2, a key regulator of JA-mediated signaling. Under stress conditions, JAZs interact with CORONATINEINSENSI-TIVE 1 (COI1) and are degraded through the SCF^COI1^/26S proteasome pathway. Degradation of JAZs activates expression of JA-response genes [50]. However, it has been proposed that JAZs also regulate other hormone pathways through interactions with hormone-related proteins such as HISTONE DEACETYLASE 6 (HDA6) [51], ETHYLENE INSENSITIVE 3 (EIN3) [51] and DELLA [52]. Thus, JAZ may be involved in crosstalk between other pathways and JA signaling [53].

There are no reports on the roles of JAZ proteins in legume-rhizobia symbiosis. However, in this study, we have identified a JAZ protein in *A*. *sinicus*, which was designated AsJAZ1. The interaction of AsJAZ1 with AsB2510 (a Lb) was verified, the expression patterns of AsJAZ1 and AsB2510 genes were examined under symbiotic conditions, and the symbiotic phenotypes of AsJAZ1-RNAi hairy roots were determined. Our results show that AsJAZ1 plays an essential role in nodule development and symbiotic nitrogen fixation.

Reduced *AsJAZ1* abundance produced by RNAi led to abnormal development of bacteroids, accumulation of poly-β-hydroxybutyrate (PHB) and a loss of nitrogenase activity. The symbiotic phenotypes of *AsJAZ1* RNAi plants were consistent with the phenotypes of *L*. *japonicus* with RNAi-mediated suppression of Lb expression reported by Ott et al. [15]. Lbs are crucial for symbiotic nitrogen fixation and involved in regulation of bacteroid and plant cell differentiation; however, the identities of molecules upstream and downstream of Lbs in such processes have not been reported. The symbiotic phenotypes of AsJAZ1 characterized in this study indicate that AsJAZ1 participates with a Lb (AsB2510) in a functional pathway regulating legume-rhizobia symbiosis, which was reported here for the first time. These results provide evidence suggesting functional association and protein interaction between AsJAZ1 and AsB2510 and thus indicated a potential regulatory role for the AsJAZ1-AsB2510 complex during nodule development and nitrogen fixation, which may be connected to the JA signaling pathway.

JA is a signaling molecule that participates in the regulation of diverse growth and development processes in plants [54–56]. In rhizobia-legume symbiosis, JA regulated nodulation and nitrogen fixation. JA is a repressor of nodulation that interferes with calcium spiking, decreases the responsiveness of root hair to Nod factors, and suppresses root elongation and nodulation in *M*. *truncatula* but does not affect *nod* gene expression in *Sinorhizobium meliloti* [57]. Similarly, methyl jasmonate applied to the shoots inhibited the formation of infection threads, *NIN* gene expression, reduced nodule number and nodule fresh weight, and suppressed nodule development in *L*. *japonicus* [58, 59]. In contrast, jasmonates induced the expression of *nod* genes in *Rhizobium leguminosarum* [60] and *Bradyrhizobium japonicum*,promoting nodulation and nitrogen fixation [61]. JA can serve as a positive or negative factor regulating nodulation and nitrogen fixation, with its effect depending on host species, the type of JA, and how and where the hormone is inoculate [62]. Given that JAZ is a key regulator of JA signaling and JA regulates nodulation and nitrogen fixation, we speculate that AsJAZ1 may participate in the regulation of nodulation and nitrogen fixation through interaction with the JA signaling pathway.

The results of this study allow some speculation with regard to the function of AsJAZ1 in symbiosis. In comparison with most other JAZ proteins, AsJAZ1 has a different subcellular localization and interacts with AsB2510 via a different binding domain (TIFY domain vs. Jas domain), suggesting that it may function differently from such proteins. Of the 2 domains characteristic of JAZ proteins, the TIFY domain mediates the homomeric and heteromeric interactions between JAZs [63]. While Pauwels et al. [64] suggested that AtJAZ1 recruited auxin signaling-related protein,TOPLESS (TPL)/TPL-related protein (TPR) via interaction with the NOVEL INTERACTOR OF JAZ (NINJA) protein through its TIFY domain. The JAZ-NINJA-TPL/TPR complex was responsible for inhibition of JA-responsive gene activation by MYC2. This work suggests that the TIFY domain also mediates the interaction between JAZs and other proteins outside TIFY family. Therefore, it is not surprising that AsJAZ1 functions by interacting with AsB2510 through its TIFY domain during symbiosis. Here, we speculate that AsB2510 in *A*. *sinicus* may be involved in the regulation of AsJAZ1 activity. AsB2510 possibly transmits symbiosis signals into the cells, inhibiting or activating AsJAZ1. AsJAZ1 interacts with proteins related to its signaling function through its Jas domain and activates or suppresses downstream targets associated with JA, acting as a regulator of symbiosis. It is worth noting that Lbs have been shown to bind NO, an important symbiotic signal molecule involving in nodule development and function [62]. Recent research has revealed that NO is involved in JA, salicylate and ethylene signaling pathway [65].

Until now, the function of JAZs in symbiotic nitrogen fixation has not been reported. Therefore, the present study provides novel insights into the function and mechanism of JAZs and Lbs during legume-rhizobia symbiosis. Future studies should focus on the downstream targets of the AsJAZ1-AsB2510 complex to reveal the signaling pathways that it participates in.

## Supporting Information

S1 Table Primer sequences for PCR. (DOCX)

S2 Table Primer sequences for GST pull-down and RNAi. (DOCX)

S1 Text Amino acid sequences of JAZ proteins for ML phylogenetic tree construction. (DOCX)
