# Supplementary material for: A JAZ Protein in Astragalus sinicus Interacts with a Leghemoglobin through the TIFY Domain and Is Involved in Nodule Development and Nitrogen Fixation
Source: PLoS One. 2015 Oct 13;10(10):e0139964. doi: 10.1371/journal.pone.0139964 (PMC4603794; doi:10.1371/journal.pone.0139964)
Supplement: S1 Table — (DOCX) [file pone.0139964.s001.docx]

**Supporting information**

**S1 Table Primer sequences for PCR**

| Primer | Sequence |
| --- | --- |
| AsJAZ1-5’race | GGCCAAAACAATCTGCAAAGGGGAT |
| AsJAZ1-3’race | GCGGATTCTAACACTGGAATGTTGG |
| AsJAZ1-forward | CGGAATTCATGTCAACTTTCCCTAATACG |
| AsJAZ1-reverse | CGGGATCCTTATAAGTTAAGGTCAAATTGCTTGGAA |
| AsJAZ1-1forward | CCGGAATTCAGGTCCCGTAAGGCACCG |
| AsJAZ1-1reverse | CGCGGATCCAGGGTTGTTGAGTTGGTAAGGTCCT |
| AsJAZ1-2forward | CCGGAATTCAGGTCCCGTAAGGCACCG |
| AsJAZ1-2 reverse | CGCGGATCCTGGATATCTCATATCTGAACCAACG |
| AsJAZ1-3forward | CCGGAATTCAGGTCCCGTAAGGCACCG |
| AsJAZ1-3 reverse | CGCGGATCCCTTGGTGGCCAATTCCATGACT |
| AsJAZ1-4forward | CCGGAATTCAGGTCCCGTAAGGCACCG |
| AsJAZ1-4 reverse | CGCGGATCCGGACCCTGTTGACCTATTTAAATTA |
| AsB2510-forward | CGCGGATCCATGGGTTTCACTGAGGCACA |
| AsB2510-reverse | CCGCTCGAGTTAACTCATAGCTTTCTTAATTTCA |
