# Supplementary material for: A JAZ Protein in Astragalus sinicus Interacts with a Leghemoglobin through the TIFY Domain and Is Involved in Nodule Development and Nitrogen Fixation
Source: PLoS One. 2015 Oct 13;10(10):e0139964. doi: 10.1371/journal.pone.0139964 (PMC4603794; doi:10.1371/journal.pone.0139964)
Supplement: S2 Table — (DOCX) [file pone.0139964.s002.docx]

**Supporting information**

**S2 Table Primer sequences for GST pull-down and RNAi**

| Primer | Sequence |
| --- | --- |
| RNAi-forword | GGGGACCTTACCAACTCAACAACC |
| RNAi-reverse | TATGAACTTGGCATTCAATATTC |
| AsJAZ1cDNA-forword | TCATAGGTTTCTTCTTGGTGG |
| AsJAZ1cDNA-reverse | TATGAACTTGGCATTCAATATTC |
| q AsJAZ1-forward | CGTGATGCAGCTGTTCAACTTCGAG |
| qAsJAZ1-reverse | CTTCCCAAGCAGCACTCAGTTCAGC |
| qAsB2510-forward | ACTGAAGCAAGTCCCTCTGTCGC |
| qAsB2510-reverse | GGTCCTCTGGCAATCACCCTATC |
