# Supplementary material for: A JAZ Protein in Astragalus sinicus Interacts with a Leghemoglobin through the TIFY Domain and Is Involved in Nodule Development and Nitrogen Fixation
Source: PLoS One. 2015 Oct 13;10(10):e0139964. doi: 10.1371/journal.pone.0139964 (PMC4603794; doi:10.1371/journal.pone.0139964)
Supplement: S1 Text — (DOCX) [file pone.0139964.s003.docx]

**Supporting information**

**S1 Text Amino acid sequences of JAZ proteins for ML phylogenetic tree construction**

>AtTIFY2A(ZML2)

MDDLHGSNARMHIREAQDPMHVQFEHHALHHIHNGSGMVDDQADDGNAGGMSEGVETDIPSHPGNVTDNRGEVVDRGSEQGDQLTLSFQGQVYVFDSVLPEKVQAVLLLLGGRELPQAAPPGLGSPHQNNRVSSLPGTPQRFSIPQRLASLVRFREKRKGRNFDKKIRYTVRKEVALRMQRNKGQFTSAKSNNDEAASAGSSWGSNQTWAIESSEAQHQEISCRHCGIGEKSTPMMRRGPAGPRTLCNACGLMWANKGAFRDLSKASPQTAQNLPLNKNEDANLETDHQIMITVANDISN

>AsJAZ1

MSTFPNTVADGRRSRKAPEKSNFSYTCTLLSQFLKEKRISADSNTGMLGKLDPKATTKDLLGNLEQNSEGALRQKVSAMDFLPQLVENPCIKKSNLNRSTGSESPQLTIFYAGKMLVFDTFAAAKATEVMELATKLASETPSTEASPSVAPVVSDKLKQSKAPQTNIALEPPRPGNQAVGSDMRYPRRASLLKFLEKRKDRVIARGPYQLNNPLKQGGSSSSGGDPEGSCSKQFDLNL

>AtJAZ11

MAEVNGDFPVPSFADGTGSVSAGLDLLVERSIHEARSTEPDASTQLTIIFGGSCRVFNGVPAQKVQEIIRIAFAGKQTKNVTGINPALNRALSFSTVADLPIARRRSLQRFLEKRRDRSTKPDGSMILPSQLTIIFGGSFSVFDGIPAEKVQEILHIAAAAKATETINLTSINPALKRAISFSNASTVACVSTADVPIARRRSLQRFFEKRRHRFVHTKPYSATTSEADKNETSPIVT

>AtJAZ12

MTKVKDEPRASVEGGCGVADGDGGAAEIGGTGSVEKSINEVRSTEIQTAEPTVPPNQLTIFFGGSVTVFDGLPSEKVQEILRIAAKAMETKNSTSISPVSSPALNRAPSFSSTSNVASPAAQPFPIQPISFCRSTADLPIARRHSLQRFLEKRRDRLVNKNPYPTSDFKKTDVPTGNVSIKEEFPTA

>AtJAZ8

MLLQQNCDLELRLFPTSYDSDSSDTTSVVESTSSGNPQPNEESQRITIFYNGKMCFSSDVTHLQARSIISIASREMKTKSSSNGSDPPNKSTSFHHNQLPNPKASMKKSLQSFLQKRKIRIQATSPYHSRR

>AtJAZ7

MIIIIKNCDKPLLNFKEMEMQTKCDLELRLLTSSYDSDFHSSLDESSSSEISQPKQESQILTIFYNGHMCVSSDLTHLEANAILSLASRDVEEKSLSLRSSDGSDPPTIPNNSTRFHYQKASMKRSLHSFLQKRSLRIQATSPYHRYR

>AtJAZ4

MERDFLGLGSKLSPITVKEETNEDSAPSRGMMDWSFSSKVGSGPQFLSFGTSQQETRVNTVNDHLLSSAAMDQNQRTYFSSLQEDRVFPGSSQQDQTTITVSMSEPNYINSFINHQHLGGSPIMAPPVSVFPAPTTIRSSSKPLPPQLTIFYAGSVLVYQDIAPEKAQAIMLLAGNGPHAKPVSQPKPQKLVHSLPTTDPPPTMPPSFLPSISYIVSETRSSGSNGVTGLGPTKTKASLASTRNNQTAAFSMAPTVGLPQTRKASLARFLEKRKERVINVSPYYVDNKSSIDCRTLMSECVSCPPAHHLH

>AtJAZ3

MERDFLGLGSKNSPITVKEETSESSRDSAPNRGMNWSFSNKVSASSSQFLSFRPTQEDRHRKSGNYHLPHSGSFMPSSVADVYDSTRKAPYSSVQGVRMFPNSNQHEETNAVSMSMPGFQSHHYAPGGRSFMNNNNNSQPLVGVPIMAPPISILPPPGSIVGTTDIRSSSKPIGSPAQLTIFYAGSVCVYDDISPEKAKAIMLLAGNGSSMPQVFSPPQTHQQVVHHTRASVDSSAMPPSFMPTISYLSPEAGSSTNGLGATKATRGLTSTYHNNQANGSNINCPVPVSCSTNVMAPTVALPLARKASLARFLEKRKERVTSVSPYCLDKKSSTDCRRSMSECISSSLSSAT

>AtJAZ9

MERDFLGLSDKQYLSNNVKHEVNDDAVEERGLSTKAAREWGKSKVFATSSFMPSSDFQEAKAFPGAYQWGSVSAANVFRRCQFGGAFQNATPLLLGGSVPLPTHPSLVPRVASSGSSPQLTIFYGGTISVFNDISPDKAQAIMLCAGNGLKGETGDSKPVREAERMYGKQIHNTAATSSSSATHTDNFSRCRDTPVAATNAMSMIESFNAAPRNMIPSVPQARKASLARFLEKRKERLMSAMPYKKMLLDLSTGESSGMNYSSTSPT

>AtJAZ10

MSKATIELDFLGLEKKQTNNAPKPKFQKFLDRRRSFRDIQGAISKIDPEIIKSLLASTGNNSDSSAKSRSVPSTPREDQPQIPISPVHASLARSSTELVSGTVPMTIFYNGSVSVFQVSRNKAGEIMKVANEAASKKDESSMETDLSVILPTTLRPKLFGQNLEGDLPIARRKSLQRFLEKRKERLVSTSPYYPTSA

>AtJAZ1

MSSSMECSEFVGSRRFTGKKPSFSQTCSRLSQYLKENGSFGDLSLGMACKPDVNGTLGNSRQPTTTMSLFPCEASNMDSMVQDVKPTNLFPRQPSFSSSSSSLPKEDVLKMTQTTRSVKPESQTAPLTIFYAGQVIVFNDFSAEKAKEVINLASKGTANSLAKNQTDIRSNIATIANQVPHPRKTTTQEPIQSSPTPLTELPIARRASLHRFLEKRKDRVTSKAPYQLCDPAKASSNPQTTGNMSWLGLAAEI

>AtJAZ2

MSSFSAECWDFSGRKPSFSQTCTRLSRYKEKKGSFGDLSLGMTCKPDVNGGSRQPTMMNLFPCEASGMDSSAGQEDIKPKTMFPRQSSFSSSSSSGTKEDVQMIKETTKSVKPESQSAPLTIFYGGRVMVFDDFSAEKAKEVIDLANKGSAKSFTCFTAEVNNNHSAYSQKEIASSPNPVCSPAKTAAQEPIQPNPASLACELPIARRASLHRFLEKRKDRITSKAPYQIDGSAEASSKPTNPAWLSSR

>AtJAZ5

MSSSNENAKAQAPEKSDFTRRCSLLSRYLKEKGSFGNIDLGLYRKPDSSLALPGKFDPPGKQNAMHKAGHSKGEPSTSSGGKVKDVADLSESQPGSSQLTIFFGGKVLVYNEFPVDKAKEIMEVAKQAKPVTEINIQTPINDENNNNKSSMVPDLNEPPTDNNHLTKEQQQQQEQNQIVERIARRASLHRFFAKRKDRAVARAPYQVNQNAGHHRYPPKPEIVTGQPLEAGQSSQRPPDNAIGQTMAHIKSDGDKDDIMKIEEGQSSKDLDLRL

>AtJAZ6

MSTGQAPEKSNFSQRCSLLSRYLKEKGSFGNINMGLARKSDLELAGKFDLKGQQNVIKKVETSETRPFKLIQKFSIGEASTSTEDKAIYIDLSEPAKVAPESGNSQLTIFFGGKVMVFNEFPEDKAKEIMEVAKEANHVAVDSKNSQSHMNLDKSNVVIPDLNEPTSSGNNEDQETGQQHQVVERIARRASLHRFFAKRKDRAVARAPYQVNQHGSHLPPKPEMVAPSIKSGQSSQHIATPPKPKAHNHMPMEVDKKEGQSSKNLELKL

>Medtr2g019190.1

MRRNCNLELCLFPPYNSSNHQNHPMVEEEEEEDSNESTPMQNQHQPLTIFYDGKMCVTDVTELQAKSILMLANRIKVQEKVMTPIGSEPSTPTTIVQSPHQLYSPGPGLSMKRSLQRFLQKRKNRVQEASPYYH

>Medtr8g021380.1

MANLTNTVSDGRRLTCKAPEKFKFSQTCSLLSQFLKEKRISGDATPSFFGKMEPKASTKDLLANMQNSDGGLRLNASAIESLPQLVENPCIKKSNTRSTDPKTPQLTIFYSGKMLVFDAFSPSNATEIMELATKLASENPSTEENPPSAPVTTEKLKESEIPQTNTALETTEQGNQAKCSDMRYPRRASLLKFLEKRKERVIARGPYQINGHKNEGSSSGSEPKDHSSDQFDLNL

>Medtr2g042900.1

MSTSSEYSEVSGNKPPAKSPEKTTFSQTCSLLSQYIKEKGCFKDLSLGITCNNNNTDPSGSSETSSQSATTMNLFPTMENNLSQKNLTTMDLLTPQAALNNSNAIKGPKAAQLTMFYNGQVIVFDDFPADKAQELMAFANKGISQSQNNSVYTYTQSQPSFPPNLVRTSVNTTTPIVPTVNIIPSTATGTGSMNEHLQVPSRPNLCDLPIMRKASLHRFLEKRKDRIAANAPYQINKPAESMSWLVGAKSTQI

>Medtr1g031930.2

MERDFLSLCSKESSPEINNEGSKNSGFSNVSAVKWPFLNKVAVHSYLTPFKVSEDDKAKMISSGFIQNINGANAKQSLLGGLPVTAPHSVLPIVGTVAGLVEPCEKPSAPAPQLTMFYGGTVNIFNDITPEKAQAIMLLAGSGLSAASNRAQPEVQASSSKFASGDDGLPISPPVYIPPCSGISSPLSVSSHTGPQPGSGSSSSDEFMAAKTSRGPTPTTSACKVVTPKVVNATTMIPSAIPQARKASLARFLEKRKERVMSTAPYNLNKKSEDAQMPNSMGANISATTGTANMLVANQG

>Medtr8g107300.1

MRRNCSNLELCLFPLYDSGNHNNSNHHIGVEEESPMQNQQQPLTIFYDGKMCLTDVTEFQAKSILMLANKIKLQEKVKTPRGSEPTTPVQSGLSMKRSLQRFLQKRKNRVQEASPYLH

>Medtr5g013530.2

MLFNYYSVMKANIKKEEPSCAQMTIFYDGKVIVFDDVPADKAKDIMDFSTKGIASTSQNHNNNYAYSSFLSRNSLQDYPQVPSIPVIYDLPMTRKASLHRFLEKRKDRIAAKAPYQTSNPAAFLNKPIDESMSWLSLAPQSECSSTSVLFL

>Medtr6g069870.1

MQWSFSNKVSNLPQFMSFKNNTHEDRSRNNVMDPVASSGYMTISTKDAFDSNQKSFLGVTQENLAIKQVGNKHGITIYPIQSSDAQSVCNQEARTISVSNQSNHVITGINMVNSVTNSQTFGSKSSATPLSVLPSKGPIVGSTDLRSRNCSKSNGTTPAQLTIFYGGTVCVYDDISPEKAKAIMLLAGNGTKMQQEISIPSKKDNFIISQPYPSPLPSPIPMTSHASTQPRGGSSSNNEVTIIRTLGHSIAKSSHNDLSHLSSLPSPLPMTSHASSYPKGGSSSNNEVTIIRSLGPSNAPTNHLESPIVATSVGLTPTNVIQPVGLPQARKASLTRFLEKRKERAMSTSPYYMSKISPECSTGSDNASFSIDFSGSSTQPPTNLPLRRTCMEVIR

>Medtr5g014515.1

MKANIIKEEPKCTQMTIFYDGKIIVFDDIPAEKVEDIMVFSSEGTTTSRNHKNNNYAFRFAQSHPSFLARNSANNSVQVPSSPVIYDLPMTRKASLHRFLEKRKDRIAAKAPYQTSNPTNLNKPINEFMSWLSLAPQIRM

>Medtr4g125960.1

MSTSSDISGLSGNKLTKSSEKPTFSQTCNLLRQYLKEKKGSFEGFNLHTPETNGSSPGSSSHSGITMDLFPTNVTPKNLTTMDFFFPRVVNPMVKEPETAQLTMFYNGQVIVLDDFPAEKVEELKSFARTQTQHSDVPTMIPQQPPSLIDMPIARKASLRRFMEKRKDRVSVYSPYQRICPDSAAPEKHAESAPWLVLGAKST

>TIFY11B[Cicer arietinum]

MSTFPNTVSDSQRSGKAPEKFKFSQTCSLLSQFLKEKRISGDSTPGLFGKIKPKASTKDLLGNKQNSDGGLRLNASAMDSLPQLVENPCIKKSNIRSTNSETPQLTIFYAGKMLVFDAFRPEKATEIMELATKLASENSSREENPTSAPITSEKLKDSKVPQPKTALETPRENQVIGSDMRYPRRASLLKFLEKRKERVISRGPYQINNHKIEGSSSGGEPKEQCSKHFDLNI

>hypothetical protein [Phaseolus vulgaris]

MSSFSNTVSEARRSGKAPEKSSFSQTCSLLSQFLKEKRASGDSTLGMVGKMEPKASTKDLFGSLQNSDGALKLSASAMDFLPQLVENPCIKKSNLSSSSVPESPQMTIFYAGKMLVFDAFPPEKATEVMELATKLASDTSGAEETPPSGPVTTKELAEAKVPQTNTSETPKLGSQGVGSDMRYPRRASLLKFLEKRKERVIARGPYQMNN

QKAEGSSSGGEPEDQCSKQFDLNL

>AFK36192.1 [Lotus japonicus]

MATFPNTVADGRSSGKALEKSNFSQTCSLLSKFLKEKRGSGDSVSGMGGKMDPKACTKDLLANLQKSDGTLRPNASSAMDILPQLVENPCIKKSNVRSAGPKTPQLTIFYAGKLLVFDGFVPEKATEVMELATKLASDSSSSEENPPKAPVVAEKLKESKAPQTNLASETSRPGNQAVRSDMRYPRRASVLKFLEKRKERVIAKGPYQVN

NPKHEGSSSGGEPEDQSSKHLDLN

>NP_001239983.1 [Glycine max]

MSSFPNTVAEGRRSGKAPEKSTFSQTCSLLSQFLKEKRASADSTLGIGGKMEPKANTKALLGSLQNSDGALKLSASAMEFLPQLVENPCIKKSRSPGPESPQLTIFYAGKMLVFDAFPPEKATEVMEMATKLASNNSGTEESPPSLPVTTEKLAVSKMPQTNTSSETPKPGNQGVGSDMRYPRRASLLKFLEKRKERVNARGPYQMNNLK

PEGSSSGGEPEDQCSKQFDLNF
